# Assessment of blood levels of heavy metals including lead and manganese in healthy children living in the Katanga settlement of Kampala, Uganda

**DOI:** 10.1186/s12889-018-5589-0

**Published:** 2018-06-08

**Authors:** Sarah E. Cusick, Ericka G. Jaramillo, Emily C. Moody, Andrew S. Ssemata, Doreen Bitwayi, Troy C. Lund, Ezekiel Mupere

**Affiliations:** 1 Department of Pediatrics, University of Minnesota, 717 Delaware Street SE, Minneapolis, MN 55414 USA; 2 Icahn School of Medicine at Mount Sinai, 1428 Madison Avenue, New York, NY 10029 USA; 3 Department of Psychology, Makerere University, Kampala, Uganda; 4 Global Health Uganda, Kampala, Uganda; 5 Department of Paediatrics and Child Health, Makerere University, Kampala, Uganda

**Keywords:** Metals, Environment, Lead, Manganese, Africa

## Abstract

**Background:**

Exposure to environmental heavy metals is common among African children. Although many of these metals are known neurotoxicants, to date, monitoring of this exposure is limited, even in countries such as Uganda that are undergoing rapid industrialization. An assessment of the burden and potential causes of metal exposure is a critical first step in gauging the public health burden of metal exposure and in guiding its elimination.

**Methods:**

In May 2016, we enrolled 100 children between the ages of 6 and 59 months living in the Katanga urban settlement of Kampala, Uganda. We measured whole blood concentrations of antimony, arsenic, barium, cadmium, cesium, chromium, cobalt, copper, lead, manganese, nickel, selenium, and zinc. Applying reference cutoffs, we identified metals whose prevalence of elevated blood concentrations was > 10%. We also administered an environmental questionnaire to each child’s caregiver to assess potential exposures, including source of drinking water, cooking location and fuel, materials used for roof, walls, and floor, and proximity to potential pollution sources such as main roads, garbage landfills, and fuel stations. We compared log-transformed blood metal concentrations by exposure category, using t-test for dichotomous comparisons and ANOVA for comparisons of three categories, using Tukeys test to adjust for multiple comparisons.

**Results:**

The prevalence of high blood levels was elevated for six of the metals: antimony (99%), copper (12%), cadmium (17%), cobalt (19.2%), lead (97%), and manganese (36.4%). Higher blood manganese was significantly associated with having cement walls (*p* = 0.04) or floors (*p* = 0.04). Cadmium was greater among children who attended school (< 0.01), and cobalt was higher among children who lived near a garbage landfill (*p* = 0.01).

**Conclusions:**

Heavy metal exposure is prevalent in the Katanga settlement and may limit neurodevelopment of children living there. Future studies are needed to definitively identify the sources of exposure and to correct potential nutritional deficiencies that may worsen metal absorption.

**Electronic supplementary material:**

The online version of this article (10.1186/s12889-018-5589-0) contains supplementary material, which is available to authorized users.

## Background

Heavy metals are ubiquitous in the environment, and the greatest burden of toxic exposure is often borne by children in low-income countries, where biomonitoring data are scant and little regulation exists for waste [1]. Many of these metals are known to be neurotoxic to developing brains, but lack of knowledge and expensive testing supplies has hindered measurement and assessment of their burden in children in low-income countries.

A growing body of evidence suggests that exposure to multiple different heavy metals and metal mixtures can have a profound neurotoxic effect [2–4]. Lead exposure is known to cause intellectual deficit, neurodevelopmental delay, and increased risk for cognitive disorders including ADHD [5]. Manganese excess causes direct damage to neuronal cells [6], while arsenic toxicity in drinking water was shown to dose dependently inhibit antioxidant activity and increase oxidative stress in the rat brain [7]. Even excess of nutritional metals such as iron and copper can cause neuronal damage [8]. Although these effects have been largely unstudied in children in low-income countries, it is in these regions where heavy metal exposure may have its most damaging neurobehavioral consequences, both independently and also via interaction with nutritional deficiency and infectious illness. While these relationships are complex, the high risk for heavy metal exposure for children in low-income countries and the known neurotoxic effects of heavy metal exposures compel monitoring of blood metal levels and examination of possible environmental determinants.

To assess the burden of heavy metal exposure in healthy Ugandan children, we recently conducted a community-based survey of blood levels of 13 heavy metals in 100 children younger than five years of age living in the Katanga urban settlement of Kampala. We also administered an environmental questionnaire to identify factors associated with metal exposure. The overall aim of the study was to determine the burden of heavy metal exposure to children in this community and to identify possible sources of exposure. A finding of prevalent metal exposure would highlight a potentially remediable cause of morbidity in this population and lay the foundation for future longitudinal studies with neurobehavioral outcomes.

## Methods

### Participants

In May 2016, we conducted a cross-sectional survey of 100 children between the ages 6 and 59 months from the Katanga community, an urban settlement near Mulago Hospital in Kampala city, Uganda. Katanga has many motor garages and car washing bays. It is situated in a low-lying drainage area for Mulago and Makerere hills. There is the open-drainage channel that connects to Nakivubo Channel. Katanga has poor garbage management systems and unprotected water sources. Most houses are makeshift temporary structures made from mud and plastic bags. With the help of a local guide, we used Global Positioning System [(GPS) Garmin eTrex 10] to map the area and to identify households with potentially eligible children, i.e., those with children between the ages of 6 and 59 months. We selected every other eligible household to approach for inclusion in the study.

Once a household was identified as eligible, we approached caregivers to determine eligibility of the child and interest to participate. A child was considered eligible if he/she was between the ages of 6 and 59 months, was a permanent resident of the Katanga settlement, and had a caretaker willing to bring the child to Mulago Hospital that day for a medical examination, blood draw, and environmental questionnaire. Any child found to require urgent medical attention [9], including any child not able to drink or breastfeed, vomiting, lethargic, unconscious, convulsing, having severe acute malnutrition, or in respiratory distress, was not eligible for the study but was instead transported immediately to Mulago Hospital for treatment. To ensure comprehension of the informed consent documents, children were also excluded from the study if their caretaker did not speak English or Luganda. If an eligible household had more than one eligible child, the caregiver selected one at random by drawing straws.

If interested in the study, caregivers and their children were escorted approximately one kilometer to Mulago Hospital. After written informed consent was obtained from the caretaker, a venous blood sample was collected from each enrolled child into a royal-blue, metal-free vacutainer tube. Malaria testing was performed with a rapid diagnostic test. Positive results were followed-up with a Giemsa blood smear. A targeted physical exam was performed, including measurement of height and weight. Finally, study staff administered an environmental questionnaire identifying possible sources of exposure to heavy metals.

The environmental questionnaire included questions assessing house construction, including floor and wall composition, presence of painted walls, roof construction; household cooking, including location of cooking, presence of windows, cooking fuel; home location, including proximity to main road, fuel station, and garbage dump; water source; and occupation of household occupants.

#### Laboratory analysis

All blood samples were refrigerated immediately upon collection, stored at 4°C onsite, and shipped to the U.S. for analysis. Blood concentrations of antimony, arsenic, barium, cadmium, cesium, chromium, cobalt, copper, lead, manganese, nickel, selenium, zinc were measured by LC-tandem mass spectrometry in the Senator Frank R. Lautenberg Environmental Health Sciences Laboratory at the Icahn School of Medicine, Mount Sinai, NY.

#### Statistical considerations

We calculated medians and percentiles of each metal to describe the distribution of values. Cutoffs to define a high value for each metal were taken from stated cutoffs (lead, cadmium, manganese, antimony, cesium, selenium) or derived from the 95% percentile of published pediatric references values for each metal [arsenic, barium, chromium, cobalt, copper, nickel, zinc (Table 1)]. Values that were below the limit of detection were assigned the value of the first significant digit of the limit of detection. The prevalence of children having a high blood level of each metal was calculated. We considered metals that had a prevalence of values above the stated threshold (Table 1) greater than 10% to be metals of concern. Because distributions of metal values were skewed, we compared log10-transformed means of each metal of concern by response category of the environmental questionnaire using a t-test for dichotomous responses and ANOVA with post-hoc Tukey’s test to account for multiple comparisons for responses with three categories.

**Table 1:** Cutoffs for blood metal levels

Heavy metal	Cutoff point	Source
Antimony	2 μg/L	Mayo Clinic [25]
Arsenic	3.12 μg/L	Goullé et al. [26]
Barium	77.6 μg/L	Goullé et al. [27]
Cadmium	0.15 μg/L	CDC [28]
Cesium	10 μg/L	NMS Labs [29]
Chromium	1.86 μg/L	Goullé et al. [26]
Cobalt	0.63 μg/L	Goullé et al. [26]
Copper	1495 μg/L	Goullé et al. [26]
Lead	2 μg/dL 5 μg/dL 10 μg/dL	CDC [28]
Manganese	18.3 μg/L	CDC [28]
Nickel	2.62 μg/L	Goullé et al. [26]
Selenium	201 μg/L	CDC [28]
Zinc	5234 μg/L	Goullé et al. [26]

## Results

Of the 100 children enrolled, there were slightly more males than females, and the average age was just over two years (Table 2**,** Additional files 1 and 2). Nearly one in four children was stunted (height-for-age Z-score < − 2). Three percent of children were wasted (weight-for-height Z-score < − 2). One child was diagnosed with and treated for *P. falciparum* malaria. Most mothers had completed upper primary school. One-third of households had a radio, 40% had a television, and more than half had electricity.

**Table 2:** Sample characteristics^a^

	100
n	
Age, months^b^	27.9 (15.1)
Sex, n (% M)	53 (53.0)
Height-for-age Z-score^c^	−0.76 (1.8)
Stunted, n (%)^c^	23 (23.5)
Weight-for-height Z^d^	−0.33 (1.0)
Wasted, n (%)^d^	3 (3.1%)
Weight-for-age Z-score^d^	−0.73 (1.2)
Underweight, n (%)	15 (15)
Malaria-positive, n (%)	1 (1.0)
Highest attained education of mother or primary caregiver^e^	
Never went to school	9 (9.1)
Lower primary school	14 (14.1)
Upper primary school	42 (42.4)
O′ level secondary school	26 (26.3)
A’ level secondary school	3 (3.0)
Tertiary level	4 (4.0)
Household possessions^e^	
Radio, n (%)	33 (33.3)
TV, n (%)	41 (41.4)
Electricity, n (%)	57 (57.6)

^a^Characterstics of 100 Ugandan children living in an urban slum near Kampala who took part in a cross-sectional survey of blood levels of heavy metals in May 2016. ^b^Mean (sd); ^c^*n* = 98; ^d^*n* = 97; ^e^*n* = 9

Heavy metal exposure was prevalent. Of the 13 metals measured, blood levels of six were elevated in this community-based sample (Table 3). All enrolled children had elevated blood antimony levels. All but three children had a blood lead level of greater than 2 μg/dL, and approximately one in three had an elevated blood manganese level. Nearly 20% had an elevated concentration of cadmium and cobalt, and more one in ten children had elevated blood copper.

**Table 3:** Blood concentrations of heavy metals in Ugandan children and prevalence of elevated levels

Metal	n	25%	50%	75%	90%	95%	Elevated, n (%)
Antimony, μg/L	100	5.71	6.61	7.29	8.31	8.76	99 (99.0)
Arsenic, μg/L	100	0.15	0.22	0.33	0.47	0.58	0 (0.0)
Barium, μg/L	100	1.00	1.44	2.42	6.27	8.29	0 (0.0)
Cadmium, μg/L	100	0.04	0.09	0.13	0.17	0.23	17 (17.0)
Cesium, μg/L	100	0.46	0.57	0.77	0.96	1.04	0 (0.0)
Chromium, μg/L	99	0.50	0.59	0.81	1.30	1.98	7 (7.1)
Cobalt, μg/L	99	0.28	0.42	0.55	0.87	1.18	19 (19.2)
Copper, μg/L	100	1025.08	1165.51	1428.08	1579.53	1719.39	12 (12.0)
Lead, μg/dL	100	4.47	5.78	7.69	10.01	10.87	3 (3.0) [0 - < 2 μg/dL] 34 (34.0) [> = 2 - < 5 μg/dL] 53 (53.0) [> = 5 - < 10 μg/dL] 10 (10.0) [> = 10 μg/dL]
Manganese, μg/L	99	9.35	13.62	23.84	31.42	37.35	36 (36.4)
Nickel, μg/L	99	0.60	0.68	0.88	1.05	1.29	1 (1.0)
Selenium, μg/L	100	106.75	121.70	151.81	167.83	181.54	1 (1.0)
Zinc, μg/L	100	3019.95	3544.39	4284.47	4946.96	5512.64	6(6.0)

The environmental questionnaire revealed some environmental factors associated with elevated blood metal concentrations (Table 4). Cement walls and floors were both associated with higher blood manganese. Children whose houses had windows tended to have higher blood antimony than children whose houses had no windows (*p* = 0.08), while children whose drinking water came from a well or spring tended to have high blood lead (*p* = 0.10) than children whose drinking water came from a community or home tap. Although only five children lived near a garbage landfill, these children had significantly higher blood cobalt than children who did not live near a landfill (*p* = 0.01). Finally, the 16 children who attended school had significantly higher blood cadmium levels than children who did not attend school (*p* < 0.01).

**Table 4:** Median whole blood heavy metal concentrations by selected environmental characteristics^1^

	n (%)	Antimony, μg/L	p^2^	Cadmium, μg/L	p	Cobalt, μg/L	p	Copper, μg/L	p	Lead, μg/dL	p	Manganese, μg/L	p
House													
Wall material^3^													
Cement	53 (55.2)	6.7 (5.8, 7.3)	0.75	0.09 (0.04, 0.14)	0.78	0.39 (0.29, 0.53)	0.84	1142 (997, 1429)	0.62	5.6 (3.9, 6.9)	0.22	17.2 (10.4, 27.1)^a^	0.04
Mud/dung	40 (43.8)	6.4 (5.5, 7.5)		0.08 (0.04, 0.13)		0.46 (0.33, 0.62)		1160 (1061, 1437)		6.1 (4.7, 8.9)		12.2 (9.4, 19.0)^b^	
Other	3 (3.1)	7.1 (6.6, 7.3)		0.11 (0.01, 0.14)		0.54 (0.21, 0.85)		1204 (945, 1260)		6.6 (3.7, 9.4)		9.0 (8.7, 20.8) ^a,b^	
Painted walls,^3^ doors, windows													
No	67 (69.8)	6.6 (5.7, 7.4)	0.56	0.09 (0.04, 0.14)	0.42	0.48 (0.31, 0.65)	0.08	1208 (1072, 1446)	0.84	6.1 (4.7, 8.8)	0.50	13.2 (9.4, 21.6)	0.87
Yes	29 (30.2)	6.6 (5.5, 7.1)		0.06 (0.03, 0.12)		0.36 (0.28, 0.45)		1107 (977, 1386)		4.5 (3.9, 5.8)		15.5 (9.3, 24.7)	
Floor material													
Cement^4^	51 (54.3)	6.6 (5.7, 7.2)	0.51	0.09 (0.04, 0.14)	0.78	0.40 (0.28, 0.52)	0.53	1135 (987, 1463)	0.39	5.6 (4.0, 6.3)	0.89	15.8 (9.3, 27.6)	0.04
Mud/ dung/clay	43 (45.7)	6.6 (5.7, 7.4)		0.08 (0.04, 0.13)		0.45 (0.32, 0.58)		1171 (1050, 1355)		6.1 (4.7, 8.9)		12.0 (9.0, 18.5)	
Windows^5^													
No	52 (67.5)	6.4 (5.5, 7.1)	0.08	0.09 (0.04, 0.14)	0.75	0.40 (0.29, 0.54)	0.77	1114 (978, 1345)	0.28	5.7 (4.1, 7.7)	0.30	12.9 (8.6, 19.7)	0.26
Yes	25 (32.5)	7.0 (6.5, 7.7)		0.08 (0.06, 0.12)		0.41 (0.31, 0.54)		1260 (1106, 1260)		6.1 (4.6, 7.2)		15.1 (10.5, 19.6)	
Water/Cooking													
Drinking water													
Community tap	66 (66.0)	6.6 (5.8, 7.5)	0.88	0.08 (0.04, 0.13)	0.59	0.40 (0.28, 0.54)	0.49	1133 (997, 1382)	0.52	5.6 (3.9, 6.4)	0.10	13.0 (9.3, 20.9)	0.36
Tap in home	18 (18.0)	6.4 (5.7, 6.8)		0.09 (0.03, 0.15)		0.41 (0.27, 0.63)		1227 (1063, 1467)		5.9 (4.7, 8.7)		13.5 (10.5, 15.8)	
Well or spring	16 (16.0)	6.6 (5.6, 7.3)		0.09 (0.05, 0.15)		0.48 (0.33, 0.55)		1492 (1092, 1493)		7.1 (5.7, 10.1)		20.2 (9.3, 30.1)	
Cooking where?^3^													
Inside	53 (55.2)	6.5 (5.7, 7.3)	0.44	0.09 (0.04, 0.14)	0.40	0.45 (0.31, 0.55)	0.93	1159 (1050, 1444)	0.22	5.8 (4.5, 7.7)	0.26	13.0 (8.7, 19.7)	0.27
Outside	43 (44.8)	6.6 (5.8, 7.3)		0.07 (0.03, 0.12)		0.41 (0.28, 0.53)		1160 (1005, 1427)		5.8 (4.4, 7.7)		15.0 (10.5, 25.3)	
Home located near													
Main road^6^													
5 m or less	32 (32.3)	6.7 (5.5, 8.2)	0.22	0.07 (0.04, 0.13)	0.66	0.44 (0.31, 0.60)	0.73	1135 (982, 1416)	0.60	6.0 (4.6, 8.1)	0.46	17.1 (9.7, 27.1)	0.10
¼ mile +	67 (67.7)	6.5 (5.7, 7.2)		0.09 (0.04, 0.14)		0.41 (0.28, 0.54)		1195 (1032, 1429)		5.7 (4.4, 7.7)		13.1 (9.0, 20.2)	
Landfill^6^													
No	94 (94.9)	6.6 (5.7, 7.3)	0.57	0.09 (0.04, 0.13)	0.88	0.41 (0.28, 0.54)	0.01	1165 (1009, 1429)	0.87	5.7 (4.4, 7.4)	0.30	13.6 (9.3, 21.6)	0.65
Yes	5 (5.1)	6.5 (6.5, 6.8)		0.06 (0.04, 0.11)		0.76 (0.39, 1.2)		1160 (1049, 1208)		7.9 (6.4, 8.9)		13.0 (12.5, 24.3)	
Fuel station^6^													
No	89 (89.9)	6.6 (5.7, 7.3)	0.97	0.08 (0.04, 0.13)	0.71	0.42 (0.27, 0.55)	0.37	1171 (1038, 1429)	0.88	5.8 (4.5, 7.7)	0.70	14.7 (9.7, 24.1)	0.12
Yes	10 (10.1)	6.7 (6.0, 7.3)		0.10 (0.02, 0.13)		0.39 (0.35, 0.45)		1055 (965, 1260)		4.7 (3.9, 6.6)		9.6 (8.6, 12.9)	
Child’s School													
Attends school?													
No	84 (84.0)	6.6 (6.8, 7.3)	0.64	0.08 (0.03, 0.12)	< 0.01	0.42 (0.29, 0.54)	0.37	1206 (1030, 1437)	0.93	5.8 (4.4, 7.3)	0.15	13.6 (9.4, 23.8)	0.94
Yes	16 (16.0)	6.4 (5.2, 6.9)		0.12 (0.08, 0.20)		0.40 (0.27, 0.51)		1100 (1018, 1232)		6.9 (4.9, 9.7)		12.9 (8.5, 24.6)	
Cigarettes													
Smoke in home^6^													
No	83 (83.8)	6.6 (5.8, 7.4)	0.25	0.08 (0.03, 0.13)	0.31	0.41 (0.28, 0.58)	0.77	1195 (1009, 1429)	0.69	5.8 (4.4, 7.7)	0.34	13.5 (9.4, 20.9)	0.94
Yes	16 (16.2)	6.3 (5.3, 6.8)		0.08 (0.06, 0.13)		0.43 (0.30, 0.50)		1124 (1041, 1429)		6.0 (4.9, 7.7)		15.0 (8.3, 26.6)	

^1^Median blood metal values by potential environmental determinant in 100 Ugandan children living in Katanga urban settlement enrolled in a cross-sectional survey; ^2^*P*-value for dichotomous comparisons and from ANOVA for comparisons among three groups, with Tukey’s test for post-hoc pairwise comparisons. All metals were log-transformed before parametric analysis; ^3^*n* = 96; ^4^*n* = 94; ^5^*n* = 77; ^6^*n* = 99

More than 96% of children lived in houses with roofs made of iron sheets; 97% of households used charcoal as the main cooking fuel; and none of the 100 children ate canned food, making assessment of the contribution of these potential pollution sources to blood metal levels impossible. Similarly, no family members worked in a printing press, metal fabrication facility, quarry/mine, or at a battery or electronics recycling facility. One parent worked at a fuel station, one at a paint factory, and one at a metal smelting facility.

## Discussion

We report a high prevalence of elevated blood levels of lead, manganese, antimony, cobalt, cadmium, and copper in a community-based sample of otherwise healthy Ugandan children living in the Katanga urban settlement of Kampala. Of concern, nearly all children had elevated blood lead and one in three had elevated blood manganese. Each of these metals has established neurobehavioral consequences, necessitating further study of the sources of exposure.

Strengths of the study include the large number of heavy metals assessed, the sampling methods used, and the environmental information collected. Although the sample is representative of the children living in Katanga, the homogeneity of the sample with regard both to common environmental exposures and the universally high values of certain metals made identifying environmental determinants difficult. Although nearly all children had elevated blood lead, none of the predictors on our questionnaire was associated with higher blood lead levels. Lead was phased out of gasoline in Uganda in 2005 [10]. One survey of blood lead among Ugandan children living near the Kiteezi landfill 12 km north of Kampala also reported a high prevalence of elevated blood lead, with 20% of children, compared to 10% in our study, having a value above 10 μg/dL [10]. Proximity to the landfill was the strongest predictor of blood lead. Water from community and home taps has been associated with high blood lead in Uganda [11], but in our study, blood lead was not significantly different among children whose drinking water came from community taps, home taps, or well/spring. Although there are currently no legally-binding controls on lead paint in Uganda [12], having painted walls, doors, or windows was also not associated with blood lead levels.

In addition to small numbers in some categories, the observed lack of difference among these categories may have stemmed from the fact that lead was universally high. Blood lead greater than 2 μg/dL has been associated with deficits in IQ, cognitive delay, and behavior [13], underscoring the necessity of identifying the sources of exposure in this population. A 2008 study in Uganda reported that water from Lake Victoria, the source of much of the tap water in Kampala, was well above the WHO threshold for lead in drinking water [11]. Soil and plants were also high in lead in the same study, with higher lead content found among plants and soil adjacent to main roads as compared to 2 km away from main roads. We did not find a difference in lead among children living adjacent to a main road versus one-quarter mile away from the main road in our study. Follow-up work that directly tests the lead content of water, air, and soil near the Katanga settlement is needed to identify the sources of exposure.

More than one third of children had high blood manganese. Manganese is an essential trace mineral, but excess manganese in children has been associated with deficits in IQ, increased risk of ADHD, and poorer verbal function [14]. Drinking water is a commonly identified source of toxic levels of manganese [15], but water source was not significantly associated with manganese concentrations in our study. Instead, living in a home with cement floors or cement walls was significantly associated with higher blood manganese as compared to walls or floors made of mud or clay. Manganese is a common ingredient in cement [16], and manganese has been reported to be in cement dust from factories in the United States [17], but cement as a cause of high blood manganese is not commonly reported in the literature. Further testing of the manganese content of the cement used in the Katanga settlement is needed.

All children had an elevated antimony level, although no environmental exposure measured predicted higher antimony levels. Antimony alloys are commonly used in batteries, sheet metal, solder, bearings, castings, ammunition, and pewter [18]. When any of these materials are smelted or recycled, antimony is released into the air. Some of this antimony then returns to the soil with rainfall, highlighting a potential source of contamination. Car exhaust is another commonly reported source of antimony exposure [19].

The five children who lived near a garbage landfill had significantly higher blood cobalt than children not living near the landfill, and the 16 children who attended school had significantly higher blood cadmium than children who did not attend school. The significance in these metal levels despite the small number children with these exposures underscores the importance of these sites, i.e., the landfill and school, as potential sources of significant heavy metal exposure. Cobalt is found in magnets, grinding and cutting tools, paints, batteries, and cobalt-coated metal from electroplating of electronics [20]. Chronic cobalt exposure can cause asthma, decreased pulmonary function, and lung fibrosis [20]. Cadmium is released into the environment from a wide range of sources, including tobacco smoking, waste incineration, and fossil fuel combustion [21]. Thus, the possible sources of exposure to each of these metals are plentiful in Katanga and in the broader Kampala area.

Like manganese, small amounts of copper are required by the body, but excess exposure can cause nose and throat irritation, vomiting and diarrhea, and liver and kidney damage [22]. Potential sources of copper are plentiful and include exposure to waste water, water from copper pipes, cooking with copper cookware, or copper-containing fungicides. It is critical to note that children in Katanga have high exposure to waste water, as there is an open drainage sewer channel in the settlement. Twelve percent of the children in our sample had a high blood copper concentration, but blood copper concentration did not differ by levels of any of the environmental factors assessed. The effects of prolonged copper exposure on child neurobehavioral development are unclear.

Our pilot study was limited by small sample size and by a sample that had similar exposures which potentially limit the applicability to other populations. Certain trends, such as increased blood lead in children who drank well/spring water and higher antimony in children whose houses had windows may have reached statistical significance with a larger sample size. Also, because some exposures, such as roofs made of iron sheets and household cooking done with charcoal, were common to nearly all children, the association of these key heavy metal sources to blood levels of metals could not be assessed in this initial study.

Nevertheless, our community-based study revealed that otherwise healthy Ugandan children have significantly high blood levels of six heavy metals. Excess exposure to each of these metals is associated with harmful health consequences, and high levels of some, notably lead and manganese, are established contributors to impaired neurobehavioral development. These neurodevelopmental deficits are increased synergistically with exposure to mixtures of multiple heavy metals. Thus, testing of the soil, air, cement, water, and paint in the Katanga settlement for metal content is needed, as are nutritional studies. Approximately 40% of Ugandan children of this age are iron-deficient [23]. A high prevalence of iron deficiency among children in the current study may up-regulate absorption of many of the divalent metals, including lead and manganese, as these metals would all be absorbed through the intestinal transport protein divalent metal transporter-1, which preferentially absorbs iron, but also transports other divalent metals [24]. Follow-up longitudinal studies with nutritional and neurobehavioral assessment before and after remediation of the identified sources of metals would demonstrate the burden of metals exposure to the health, nutritional status, and long-term development of children living in Katanga.

## Conclusions

In conclusion, we found high blood levels of multiple neurotoxic heavy metals in a community-based sample of children living in the Katanga area of Kampala, Uganda. Increased industrialization of Uganda is representative of similar growth throughout sub-Saharan Africa and most low-income countries worldwide. One unintended product of this growth is unregulated industrial pollution, poor air quality, and unchecked contamination of soil, water, and air. This contamination inevitably leads to greater exposure of environmental heavy metals, potentially limiting the developmental trajectory of many African children.

## Additional files

Additional file 1: Study dataset. (XLSX 283 kb)

Additional file 2: Data codebook. (XLSX 37 kb)

## References

[1] World Health Organization and UNEP. Environment and health in developing countries. http://www.who.int/heli/risks/ehindevcoun/en/ Accessed 11 Jan 2018.

[2] Sanders AP, Henn BC, Wright RO. Perinatal and Childhood exposure to cadmium, manganese, and metal mixtures and effects on cognition and behavior: a review of recent literature. Curr Environ Health Rep. 2015;(3):284–94. 10.1007/s40572-015-0058-8. 10.1007/s40572-015-0058-8 PMC4531257 26231505

[3] Andrade VM, Aschner M, Marreilha Dos Santos AP (2017). Neurotoxicity of Metal Mixtures. Adv Neurobiol.

[4] Karri V, Schuhmacher M, Kumar V (2016). Heavy metals (Pb, cd, as and MeHg) as risk factors for cognitive dysfunction: a general review of metal mixture mechanism in brain. Environ Toxicol Pharmacol.

[5] Davis JM, Svendsgaard DJ (1987). Lead and child development. Nature.

[6] Yin L, Dai Q, Jiang P, Zhu L, Dai H, Yao Z, Liu H, Ma X, Qu L, Jiang J. Manganese exposure facilitates microglial JAK2-STAT3 signaling and consequent secretion of TNF-a and IL-1β to promote neuronal death. Neurotoxicology. 2017; 10.1016/j.neuro.2017.04.001. 10.1016/j.neuro.2017.04.001 28385490

[7] Jomova K, Jenisova Z, Feszterova M, Baros S, Liska J, Hudecova D (2011). Arsenic: toxicity, oxidative stress and human disease. J Appl Toxicol.

[8] Madsen E, Gitlin JD (2007). Copper and iron disorders of the brain. Annu Rev Neurosci.

[9] World Health Organization*.* Handbook: IMCI integrated management of childhood illness. Department of Child and Adolescent Health and Development (CAH), 2005 http://apps.who.int/iris/bitstream/10665/42939/1/9241546441.pdf. Accessed 11 Jan 2018.

[10] Graber LK, Asher D, Anandaraja N, Bopp RF, Merrill K, Cullen MR, Luboga S, Trasande L (2010). Childhood lead exposure after the phaseout of leaded gasoline: an ecological study of school-age children in Kampala. Uganda Environ Health Perspect.

[11] Mghweno LR, Makokha AO, Magoha HS, Wekesa JM, Nakajugo A (2008). Environmental lead pollution and food safety around Kampala City in Uganda. J Appl Biosci.

[12] World Health Organization. Global Health Observatory Data. http://www.who.int/gho/phe/chemical_safety/lead_paint_regulations/en/. Accessed 11 Jan 2018.

[13] Lanphear BP, Dietrich K, Auinger P, Cox C (2000). Cognitive deficits associated with blood lead concentrations. Public Health Rep.

[14] Roels HA, Bowler RM, Kim Y, Claus Henn B, Mergler D, Hoet P (2012). Manganese exposure and cognitive deficits: a growing concern for manganese neurotoxicity. Neurotoxicology.

[15] Bjørklund G, Chartrand MS, Aaseth J (2017). Manganese exposure and neurotoxic effects in children. Environ Res.

[16] Duoqiang L, Feng Q, Xuguang L, Jibo J (2011). Performance of concrete made with manganese slag. Adv Mater Res.

[17] Ogunbileje JO, Sadagoparamanujam VM, Anetor JI, Farombi EO, Akinosun OM, Okorodudu AO (2013). Lead, Mercury, cadmium, chromium, nickel, copper, zinc, calcium, iron, manganese and chromium (VI) levels in Nigeria and United States of America cement dust. Chemosphere.

[18] Antimony CAS#7440–36-0. Agency for Toxic Substances and Disease Registry. September 1995. https://www.atsdr.cdc.gov/toxfaqs/tfacts23.pdf. Accessed 11 Jan 2018.

[19] Scottish Environment Protection Agency. Antimony. http://apps.sepa.org.uk/spripa/Pages/SubstanceInformation.aspx?pid=98. Accessed 15 May 2018.

[20] Cobalt. The National Institute for Occupational Safety and Health. https://www.cdc.gov/niosh/topics/cobalt/default.html#socialMediaShareContainer Accessed 11 Jan 2018.

[21] World Health Organization. Exposure to cadmium: a major public health concern. http://www.who.int/ipcs/features/cadmium.pdf Accessed 11 Jan 2018.

[22] Copper. CAS 7440–50-8. Agency for Toxic Substances and Disease Registry. https://www.atsdr.cdc.gov/substances/toxsubstance.asp?toxid=37 Accessed 18 Jan 2018.

[23] World Health Organization. Vitamin and Mineral Information System. WHO Global Database on Anaemia. http://who.int/vmnis/anaemia/data/database/countries/uga_ida.pdf?ua=1 Accessed 11 Jan 2018.

[24] Illing AC, Shawki A, Cunningham CL, Mackenzie B (2012). Substrate profile and metal-ion selectivity of human divalent metal-ion Transporter-1. J Biol Chem.

[25] Antimony, Blood. c1995–2017. Rochester (MN): Mayo Clinic Mayo Medical Laboratories; [accessed 2 June 2017]. http://www.mayomedicallaboratories.com/test-catalog/Clinical+and+Interpretive/64273

[26] Goullé JP, Le Roux P, Castanet M, Mahieu L, Guyet-Job S, Guerbet M (2015). Metallic profile of whole blood and plasma in a series of 99 healthy children. J Anal Toxicol.

[27] Goullé J, Mahieu L, Castermant J, Neveu N, Bonneau L, Lainé G, Bouige D, Lacroix C (2005). Metal and Metalloid multi-elementary ICPMS validation in whole blood, plasma, urine and hair Reference values. Forensic Science International.

[28] CDC. Fourth National Report on Human Exposure to Environmental Chemicals Updated Tables, January 2017, Volume 1. https://www.cdc.gov/exposurereport/index.html. Accessed 11 Jan 2018.

[29] Test Summary Sheet for Cesium, Blood. Willow Grove (PA): NMS Labs; [Accessed 3 June 2017].

